# Transposable elements as scaffold/matrix attachment regions: shaping organization and functions in genomes

**DOI:** 10.3389/fmolb.2023.1326933

**Published:** 2024-02-22

**Authors:** Rashmi Upadhyay Pathak, Kundurthi Phanindhar, Rakesh K. Mishra

**Affiliations:** ^1^ CSIR-Centre for Cellular and Molecular Biology, Hyderabad, India; ^2^ Academy of Scientific and Innovative Research (AcSIR), Ghaziabad, India; ^3^ Tata Institute for Genetics and Society, Bengaluru, India

**Keywords:** nuclear matrix, nuclear matrix attachment region, transposable element, genome, topologically associated domain

## Abstract

The hierarchical structure of eukaryotic genomes has regulatory layers, one of them being epigenetic “indexing” of the genome that leads to cell-type-specific patterns of gene expression. By establishing loops and defining chromatin domains, cells can achieve coordinated control over multi-locus segments of the genome. This is thought to be achieved using scaffold/matrix attachment regions (S/MARs) that establish structural and functional loops and topologically associating domains (TADs) that define a self-interacting region of the genome. Large-scale genome-wide mapping of S/MARs has begun to uncover these aspects of genome organization. A recent genome-wide study showed the association of transposable elements (TEs) with a significant fraction of S/MARs, suggesting that the multitude of TE-derived repeats constitute a class of anchorage sites of chromatin loops to nuclear architecture. In this study, we provide an insight that TE-driven dispersal of S/MARs has the potential to restructure the chromosomes by creating novel loops and domains. The combination of TEs and S/MARs, as elements that can hop through the genome along with regulatory capabilities, may provide an active mechanism of genome evolution leading to the emergence of novel features in biological systems. The significance is that a genome-wide study mapping developmental S/MARs reveals an intriguing link between these elements and TEs. This article highlights the potential of the TE–S/MAR combination to drive evolution by restructuring and shaping the genome.

## Introduction

Genomic function is regulated by the hierarchical organization of chromatin in a 3D nuclear context. The current idea of the functional organization of chromatin in an interphase nucleus is dominated by the view that the chromatin folds into clusters of loops and segregates into two distinct types of compartments, transcriptionally repressive and permissive. At the chromosome level, the self-associating chromatin loop clusters, termed TADs, ensure cell-type specific expression of associated genes. The chromatin loops topologically couple the *cis*-regulating elements (CREs), such as the enhancers and promoters of target genes. The CREs themselves are also involved in the folding of the chromatin fiber by virtue of networking of *trans*-acting protein factors (TFs) bound to them (Szabo et al., 2019).

S/MARs (scaffold/matrix attachment regions) are DNA elements instrumental in the formation of chromatin loops as they attach to the nuclear matrix (NuMat) in the interphase nucleus. Traditionally, S/MARs have been viewed as regions of DNA that anchor chromatin loops to a proteinaceous scaffold and are thought to have a structural role. Taking cues from experimentally characterized S/MARs from the eukaryotic genome, several functions, including the initiation of DNA replication, transcriptional activation, chromatin remodeling, and insulation of domains, are attributed to S/MARs. These elements, therefore, not only act as structural loop anchors but also regulate the functional aspects of chromatin (Liebich et al., 2002). However, the functional heterogeneity, lack of conservation of DNA sequences, and difficult and method-specific experimental output have put S/MARs on the backstage of recent scientific advances. We present here an argument that these important determinants of structural chromatin loops and functional domains need to be investigated at the genomic scale in a variety of eukaryotes as they may also be drivers of genome evolution.

S/MARs are an intriguing class of CREs. Typically, CREs are non-coding DNA that are responsible for the activation and sustenance of the transcriptional status of a locus and contain binding sites for TFs and other regulatory molecules. Promoters, enhancers, and insulators are the best-understood types of CREs. Occurring exclusively in the eukaryotic genome, S/MARs are DNA elements that are defined biochemically as opposed to other CREs that are defined functionally. They are demarcated based on their attachment to a ribo-proteinaceous scaffolding structure in the nucleus known as the NuMat. Identified for the first time in *Drosophila*, these sequences have heterogenous functions and lack sequence conservation, although they have a functionally relevant conservation of the secondary structure of DNA. The reason for the sequence heterogeneity is attributed to their involvement in multiple independent processes (Mirkovitch et al., 1984; Bode et al., 1992; Laemmli et al., 1992).

A recent study indicated that TE-derived sequences identify as a significant portion of S/MARs (Sureka et al., 2022). The observation that TEs can act as S/MARs has been reported earlier as well. As TEs are mobile DNA elements with sequence properties uniquely suited to gene regulatory functions, their combination with S/MARs presents a tantalizing possibility of driving evolution through changes in domain architecture. It is well known that through diverse invasion strategies, TEs occupy a substantial fraction of all eukaryotic genomes. Environmental and genetic factors further modulate and produce dramatic variation in TE content. These factors present TEs as a major source of genetic variation and a driver of evolution (Rebollo et al., 2012; Wells and Feschotte, 2020). In the present article, we focus on the TE–S/MAR combination as powerful mobile regulatory sequences that can connect with nuclear architecture and significantly impact the evolution of genomes.

## Genome-wide distribution of S/MARs: dynamic and constitutive S/MARs

As S/MARs are implicated in the structural delimitation of chromatin loops, it is reasonable to assume that they would correlate with TADs to organize chromatin in discrete neighborhoods with coordinated gene expression. In recent years, with the advent of conformation capture techniques, there has been significant advancement in the knowledge of genome-wide chromatin topology and domain organization. However, S/MARs have never been placed in the context of chromosome folding at the genome level, mainly because information on *in vivo* S/MARs in an identical context where TADs have been mapped is not available. A couple of studies where S/MARs and TADs have been mapped in an identical setup do show that the two features coincide. The overlap of TAD borders with S/MARs in pericentromeric heterochromatin was found to be highly significant, indicating their plausible role in genome organization along with TADs (Saha et al., 2020; Verma et al., 2021). It is pertinent here to note that while TADs are generally erased during mitosis, chromatin loop anchors are thought to persist. This raises the intriguing possibility that a fraction of S/MARs (those that coincide with major TAD borders) are retained on condensed mitotic chromosomes to transmit the memory of TADs through the cell cycle. However, this aspect remains to be investigated.

Most experimentally identified S/MARs have been reported on a case-by-case basis, and genomic approaches are only beginning to emerge (Table 1). A handful of studies investigate S/MARs on the genomic scale (whole genome or whole chromosome), and only few among them experimentally map S/MARs to validate. Most studies use the bioinformatics approach as experimental mapping of S/MARs requires substantial efforts and can generate method-specific results. The bioinformatics approaches, although informative, are based on the occurrence of certain motifs (ORI sequences, TG-richness, Topo II sites, and AT-richness) or physical properties of the DNA sequence (duplex destabilization, triple helix, and curving/kinking of DNA). Severe discordance exists between sequence-based S/MAR prediction and their *in vivo* mapping, as the tools detect only a fraction of the elements experimentally mapped (Pathak et al., 2014). This is understandable as a predicted S/MAR might not be functional *in vivo* and *vice versa*. However, these observations underline the importance of mapping S/MARs *in vivo* to refine bioinformatics approaches for the purpose. In this context, robust biochemical methods for the isolation of S/MARs have proved to be very useful. Most protocols employed to isolate the NuMat are optimized to remove soluble proteins and the bulk of chromatin without aggregation to reveal the non-chromatin nuclear skeleton. The initial protocol used to isolate the NuMat employed high-salt (1 M and 2 M NaCl) extraction (Berezney and Coffey, 1974). In 1982, a modified method was introduced that used ammonium sulfate at lower molarity [0.25 M (NH_4_)_2_SO_4_] but nearly the same ionic strength as 1 M NaCl (Capco et al., 1982). Later on, concerns about the high ionic strengths involved in extraction protocols led to two alternative procedures. While Mirkovitch et al. (1984) exposed the nuclei to a low concentration of lithium 3,5-diiodosalicylate (25 mM LIS) to obtain the NuMat preparation, Jackson and Cook agarose-encapsulated the cells and electrophoretically removed the non-NuMat material (Jackson and Cook, 1986). All the alternative methods yielded NuMat preparations that ultra-structurally resembled the conventional one. However, subtle differences were noted; for instance, the non-chromatin scaffold that resists low-salt extraction (using LIS) showed better preservation of components of the lamina, nucleolus, and fibro-granular internal meshwork. In recent years, S/MARs isolated by different methods have been sequenced and compared. One such study shows that though distinct groups of elements are isolated by low-salt and high-salt extraction protocols, there is considerable overlap among them. Both methods of extraction uncover interactions that are the components of the same network, validating either the low-salt (LIS) or high-salt (NaCl) method as good enough to isolate S/MARs (Linnemann et al., 2009). Another recent study found that interventions like heat stabilization strengthen the NuMat, where the basic molecular components remain unchanged (Sureka et al., 2022).

**TABLE 1:** Genome-wide studies on S/MARs.

S. no.	Year	Organism	Approach	Key feature	Reference
1	2004	*Arabidopsis*	*In silico* mapping using SMARTest	First genome-wide investigation of S/MARs	Rudd et al. (2004)
2	2007	*C. elegans*	*In silico* computational prediction	Genome-wide analysis of the distribution of matrix recognition signature	Anthony and Blaxter (2007)
3	2007	Human	Bioinformatics computational prediction	Genome-wide prediction of MARs	Girod et al. (2007)
4	2009	Human HeLa cells	Array-based hybridization	Tiling microarray to map S/MARs on chr14 to chr18	Linneman et al. (2009)
5	2009	Human primary cell AoAF	Array-based hybridization	Tiling microarray to map S/MARs on chr14 to chr18	Linneman and Krawetz (2009)
6	2010	*Giardia*	*In silico* computational prediction	First report of S/MARs from a unicellular eukaryote, the protozoan parasite *Giardia lamblia*	Padmaja et al. (2010)
7	2014	*Arabidopsis*	Array-based genomic hybridization	Tiling microarray to map S/MARs on chr4	Pascuzzi et al. (2014)
8	2014	*Drosophila*	Biochemical isolation and NGS	First genome-wide *in vivo* mapping of MARs by sequencing	Pathak et al. (2014)
9	2019	Human	Bioinformatic data mining approach	Genome-wide mapping of S/MARs based on the ChIP-Seq data of S/MARBPs	Narwade et al. (2019)
10	2022	*Bombyx mori*	Biochemical isolation and NGS	Genome-wide mapping and dynamics of S/MARs during silk gland development	Chunduri et al. (2022)
11	2022	*Drosophila*	Biochemical isolation and NGS	Structural and developmental dynamics of S/MARs	Sureka et al. (2022)

**FIGURE 1:**
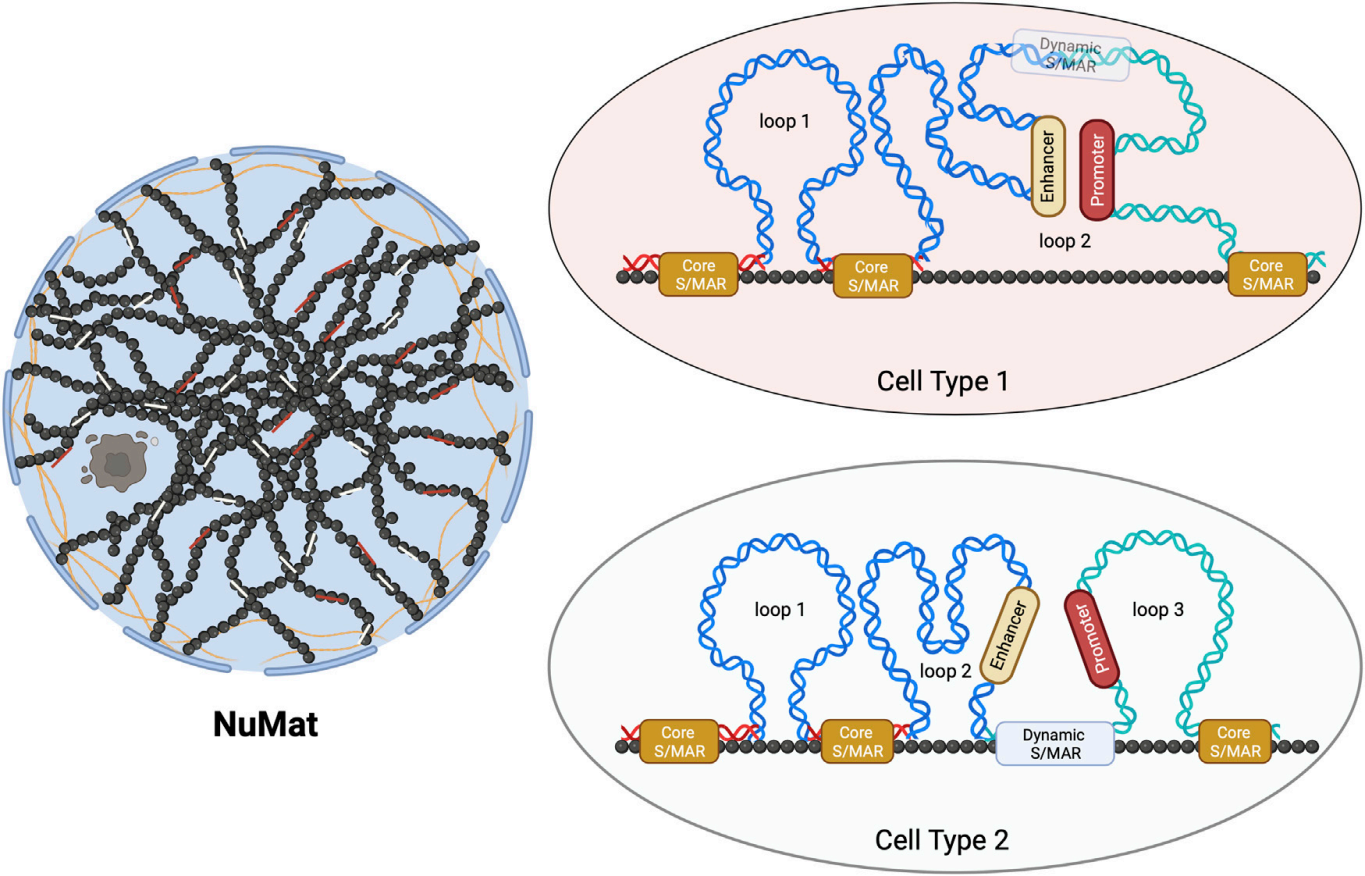
Schematic representation of Core- and dynamic S/MARs. The NuMat is the residual nuclear structure that remains after the extraction of chromatin and soluble nuclear proteins. It includes the nuclear envelope, lamina, remnants of the nucleolus, and internal ribo-proteinaceous meshwork. The base of a chromatin DNA loop that is attached to the NuMat in an intact nucleus for a function (such as transcription and replication) or for defining a loop domain evades extraction and gets isolated as an S/MAR. Core-MARs are the anchor points that remain unchanged across cell types, while dynamic S/MARs are functionally derived and cell-type-specific. As seen in the schematic, NuMat association of a dynamic S/MAR in cell type 2 results in the creation of loop 3. This association separates a promoter from its enhancer, leading to alteration in the transcriptional output.

Only two studies of genome-wide mapping of S/MAR investigate their dynamics during embryonic development or cellular differentiation (Chunduri et al., 2022; Sureka et al., 2022). These studies, for the first time, uncovered that these attachment points can be categorized as dynamic or stable (Core-MARs) (Figure 1). The idea of categorization of S/MARs is not novel and has been suggested in several previous studies. Avramova et al. (1995), while studying these elements in maize, stated that “strong” S/MARs delimit the structural domains containing the transcriptional units and the “weak” S/MARs cause dynamic folding of larger loops into subdomains. Tikhonov et al. (2000) divided these elements into two groups: the “durable” S/MARs, mapping outside genes and defining borders of chromatin loops, and the “unstable” S/MARs with regulatory roles, mapping mainly within introns. They conclude that S/MARs possess both domain-defining and regulatory roles.

With the genome-wide study conducted during *Drosophila* embryonic development (Sureka et al., 2022), it is clear that the functional contribution of S/MARs to gene regulation is driven by both dynamic and stable attachments. The dynamic attachment sites are reversible (likely to be protein factor-induced), functionally derived, closer to or within genes, and differ in various cell types. The stable “Core-MARs” are constitutive structural domains defining anchor points that remain unchanged across cell types. The interesting finding that this study brought to light is that the majority of Core-MARs are associated with repetitive DNA and that the TEs of LINE and LTR types are predominant among them. This study, for the first time, provided a glimpse into how dynamic and stable S/MARs can differ in their sequence composition and that TEs form a major class of Core-MARs. Thus, the study has identified a class of S/MARs stable across cell types with sequence characteristics different from those of the AT-rich or TG-rich classes of S/MARs containing TE-derived repeats.

## Conservation of MARs through evolution

In the absence of sequence conservation, it is difficult to study the status of S/MARs as regulatory elements across species. During evolution, segments of genomes are shuffled (by recombination), placing orthologous genes from closely related species in very different genomic environments. Such genes still show a highly similar level of transcriptional output. A hypothesis to explain this paradox is based on the location of S/MARs, as these elements would stably anchor and appropriately position genes within the nuclear space. To gain insight, S/MARs from the genomes of closely related species have been investigated. Although not conducted on the genomic scale, these studies provide valuable details of the mechanistic paradigm of S/MARs. In one such study, the *Sh2/A1*-homologous regions of two grass species, rice and sorghum (about 50 kb of sorghum and 30 kb of rice DNA), were screened for the location and distribution of S/MARs. The position of S/MARs relative to the neighboring genes was maintained, indicating the preservation of structural organization and folding of the chromatin in the region, but no conservation of sequences or motifs was found. The gene composition, orientation, order, and placement of S/MARs were found to be evolutionarily conserved in these closely related species. Interestingly, however, S/MARs in the region co-localize with TEs (Avramova et al., 1998).

In a similar study, collinear chromosomal segments of maize and sorghum at the alcohol dehydrogenase (*Adh1*) locus carrying four genes were analyzed, and S/MARs were found to be at comparable positions in the two species, often flanking individual genes. Here too, TEs were found on the same fragments as S/MARs, suggesting that TEs may themselves be the S/MARs (Tikhonov et al., 2000). Another comparison between maize and Arabidopsis *Adh* locus found similar organizational conservation, and the authors conclude that stable S/MARs create large organizational domains that are conserved, and these are distinct from the smaller functional domains created by dynamic S/MARs (Paul and Ferl, 1998).

S/MAR organization has been shown to be conserved at the orthologous loci in animal genomes as well. A study examining the interspecies conservation of looping elements at the important human imprinting center at 15q11-q13 found that despite extensive divergence of the DNA sequence, the homologous mouse locus had a similar organization of S/MARs (Greally et al., 1999). Non-coding DNA in the human–mouse orthologous intergenic regions contain “islands” of conserved sequences, a significant fraction of which were predicted S/MARs (Glazko et al., 2003). Taken together, these studies indicate that S/MAR organization is strikingly similar around an orthologous gene or group of genes, although sequence similarity may be minimal. These underline the importance of S/MARs in the evolutionary conservation of a functional genomic arrangement.

## Significance of TEs as Core-MARs

**FIGURE 2:**
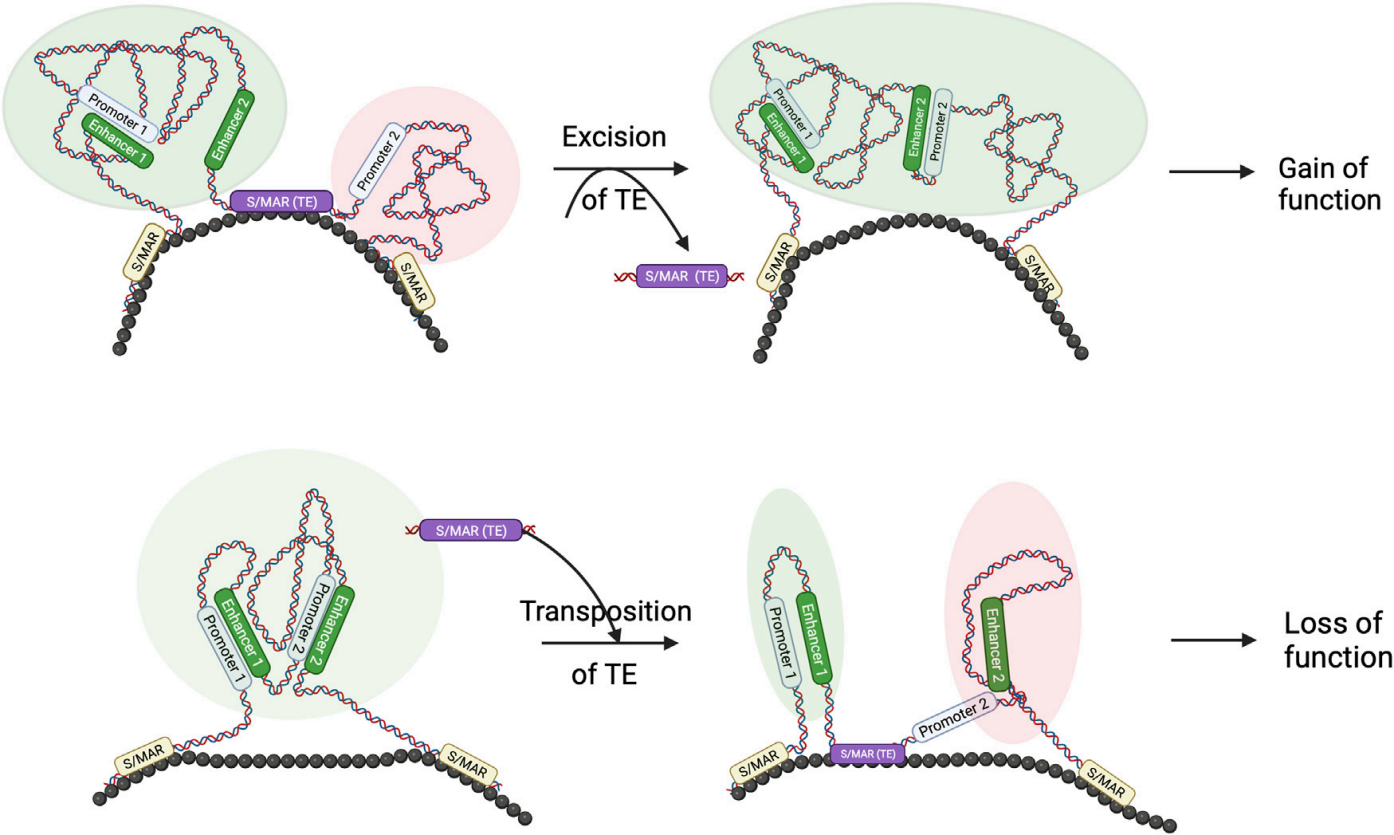
TE and S/MAR combinations can result in novel features in the genome: schematic to show that the transposition of TE and S/MAR combinations may result in architectural changes. As seen in the figure, excision of a TE, which also has S/MAR features, may lead to novel enhancer–promoter interactions, resulting in a gain of function. Similarly, hopping in of a TE–S/MAR combination may create a separate loop domain that separates a promoter from its enhancer and results in a loss of function. Such architectural changes may be evolutionarily relevant.

Although more genome-wide studies in related species are required, it is highly probable that Core-MARs are conserved to convey architectural information, and TEs form a sizeable proportion of such elements. We hypothesize that changes in architecturally placed TEs, *viz.*, Core-MARs, are events that establish specific new genome architectures that are important from the evolutionary perspective (Figure 2). The concept that genome architecture is formatted by TEs is not new, and it has been known for a while that transpositional changes cause evolutionary diversification by conferring adaptive benefits or reproductive isolation. Comparative whole-genome sequencing has provided evidence for TEs as organizers of functional genomic architecture, as these elements often map in a similar position and identical orientation in aligned syntenic segments of related genomes, indicating positive selection (Zhu et al., 2003). To get an integrated view of S/MARs in evolution, it may be worthwhile to search for cases where TEs that have been major engines of speciation are also Core-MARs.

It is also important to note here that non-coding repetitive DNA is far more taxonomically discriminating than coding sequences. For example, each order of mammals has its own characteristic set of TEs (Platt et al., 2018). Approximately 50% of the human genome is composed of TE-derived sequences compared with ∼1% of protein-coding sequences. This figure might be higher as many TE sequences in the genome would have evolved beyond recognition. The staggering evolutionary success of TEs, as evidenced by the sheer abundance of these elements in the genomes of highly evolved species, as well as the variety of changes induced by their mobility, suggest that these elements are “domesticated” to serve the evolutionary interests of their hosts. The property of TEs that enables them to provide ready-to-use mobile CREs is exploited by the host genome, and thus, TE-derived promoters, enhancers, and insulators are common (Jordan et al., 2003). TEs are also known to facilitate the partitioning of the genome into distinct domains, and this is precisely the same function envisaged for S/MARs. Notably, binding sites for CTCF (architecturally important protein, also known to bind to S/MARs) were propagated through TE expansion (Schmidt et al., 2012). Several conserved, robust, and tissue-independent CTCF sites were identified in the study that spanned six mammalian species. These were ChIP-seq data and did not investigate TADs or S/MARs in the context. However, given the properties of CTCF-binding sites, the data can be extrapolated to evolutionarily conserved TADs and Core-MARs, highlighting the link between TEs, S/MARs, and TADs through evolution.

TEs, as Core-MARs, gain more power when they land in a region of the genome that is potentially important for the regulation of a cluster of genes. Such locus control regions (LCRs) contain a complex collection of enhancer and silencer elements and confer epigenetic regulation of the linked genes in the locus. Many times, the LCRs contain abundant TE-derived sequences and S/MARs. Some of the best-characterized LCRs include the ones located at the human *β*-globin locus, MHC locus, and serpin gene locus. Importantly, each of these loci harbors several S/MARs that contain predominantly TE-derived repetitive DNA sequences, pointing to a link between the evolution of these loci and S/MARs (Rollini et al., 1999; Zhang and Qian, 2002; Ottaviani et al., 2008).

Put together, genetic signals, such as S/MARs, format the DNA for interaction with the nuclear architecture and ensure proper access to coding sequences. Among these, the Core-MARs that are lineage-specific confer a characteristic genome architecture independent of the coding sequence. In addition, as they are located in the non-coding regions, they are amenable to meaningful evolutionary changes. A subset of these elements, the combination of TEs and Core-MARs, may provide a specific mechanism for the generation of evolutionarily significant variations in the genome via changes in the domain architecture.

## MAR/TE combinations as hotspot of evolutionary events

Apart from single-nucleotide changes, large-scale rearrangements such as inversions, translocations, and fusion drive chromosome evolution. Studies reveal that chromosome breakpoints cluster in an area rich in repeats, S/MARs, and Topo II binding sites, indicating that the loop anchorage regions work as recombination hotspots (dela Paz et al., 2012). Mysterious clustering of fragile sites around S/MARs indicates that the chromatin structure around loop anchorage regions induces breakpoints that may be mediated by Topo II present on the site (Razin and Iarovaia, 2005). Interestingly, S/MARs often are hotspots for retroelement insertions (Narwade et al., 2019). As TEs naturally possess CREs, their insertions result in genome-wide dispersal of regulatory elements. At times, TEs may also carry cell-type-specific elements, adding a new regulatory property to the domain where they get inserted. The combination of TE and S/MAR insertions provides ready-to-use regulatory elements that can alter the properties of a chromatin domain, increasing the genomic reservoir that can be acted upon by natural selection. Any nonfatal transposition event retained in the genome could increase the possibilities of genetic drift and evolution. Thus, TE–S/MAR combinations may act as regions important for the restructuring of the genome during evolution. By studying TE–S/MAR in related species, it may also be possible to decipher the evolutionary record of architectural transformations.

## Conclusion

S/MARs, as dispersed regulatory sequences, offer several potential functional attributes. Not only do they connect unlinked coding sequences into co-ordinately controlled subsystems by virtue of attachment to nuclear architecture but they can also act as drivers of chromosomal changes that are evolutionarily significant. The combination of S/MARs and TEs allows TE-mediated hopping of S/MARs that brings about the reformatting of topological and functional domains, a potentially valuable component of the evolutionary process. In this context, genome-wide sequencing of *in vivo* S/MARs from various sources is highly desirable. Understanding the dynamics of these elements through the cell cycle, tissue differentiation, and organismal development would help better define their role in the organization of chromatin. Placing them alongside other relevant chromatin landmarks such as TAD borders, enhancers, and promoters will help us to understand the variety of structural and functional roles played by them. Extensive genome-wide mapping of S/MARs from several closely related species will help us to better understand the functional and evolutionary relevance of these unique regulatory elements.

## Data Availability

The original contributions presented in the study are included in the article/Supplementary material; further inquiries can be directed to the corresponding author.
